# Responsible Data Governance of Neuroscience Big Data

**DOI:** 10.3389/fninf.2019.00028

**Published:** 2019-04-24

**Authors:** B. Tyr Fothergill, William Knight, Bernd Carsten Stahl, Inga Ulnicane

**Affiliations:** Centre for Computing and Social Responsibility, School of Computer Science and Informatics, Faculty of Computing, Engineering and Media, De Montfort University, Leicester, United Kingdom

**Keywords:** data governance, human brain project, ethics, neuroscience, big data, RRI

## Abstract

Current discussions of the ethical aspects of big data are shaped by concerns regarding the social consequences of both the widespread adoption of machine learning and the ways in which biases in data can be replicated and perpetuated. We instead focus here on the ethical issues arising from the use of big data in international neuroscience collaborations. Neuroscience innovation relies upon neuroinformatics, large-scale data collection and analysis enabled by novel and emergent technologies. Each step of this work involves aspects of ethics, ranging from concerns for adherence to informed consent or animal protection principles and issues of data re-use at the stage of data collection, to data protection and privacy during data processing and analysis, and issues of attribution and intellectual property at the data-sharing and publication stages. Significant dilemmas and challenges with far-reaching implications are also inherent, including reconciling the ethical imperative for openness and validation with data protection compliance and considering future innovation trajectories or the potential for misuse of research results. Furthermore, these issues are subject to local interpretations within different ethical cultures applying diverse legal systems emphasising different aspects. Neuroscience big data require a concerted approach to research across boundaries, wherein ethical aspects are integrated within a transparent, dialogical data governance process. We address this by developing the concept of “responsible data governance,” applying the principles of Responsible Research and Innovation (RRI) to the challenges presented by the governance of neuroscience big data in the Human Brain Project (HBP).

## Introduction

The advancement of neuroscience is of critical global importance, with immense potential benefits for society. Large neuroscience projects such as the US BRAIN Initiative, Japan Brain/MINDS, and the European Human Brain Project (HBP) harness the increasing capacity of technological infrastructure to address a wide range of brain-related medical concerns, definitional questions such as what qualifies as a diseased brain, and fundamental queries like the nature of consciousness. In the case of the HBP, a 10-year Future and Emerging Technologies Flagship funded by the European Commission, developing an information technology infrastructure for continued, future neuroscience innovation is also an essential goal (Amunts et al., 2016). In pursuit of these aims, neuroscience research is becoming increasingly globalised, and progressively reliant on knowledge exchange and data sharing (Choudhury et al., 2014). The establishment of the International Brain Initiative at the end of 2017, a partnership consisting of the largest brain projects, is evidence of the high value placed on global cooperation in neuroscience. Accompanying these needs for intellectual engagement with the broader neuroscience community, increased technological reliance, and a focus on continuing innovation for the benefit of humanity is the use of big data and related analytical techniques. These analytics include descriptive, diagnostic, predictive, and prescriptive varieties, each with distinctive applications (Chalcraft, 2018). Such approaches to big data offer many potential benefits, including economic viability and the potential discovery of unanticipated trends or correlations. Big data analytics in biomedical contexts are perceived as an evolving necessity, sometimes framed as a change from hypothesis-driven research to data-driven research, and often viewed as leading to highly desirable outcomes such as targeted therapeutics and personalised medicine (Merelli et al., 2014).

Whilst big data analytics promise increased advancement of scientific understanding, the complexity of big data presents a range of technical and philosophical challenges. Furthermore, these challenges are deeply contextual, differing across disciplinary, institutional, and national boundaries and as contingent technological and ethical frameworks change over time. New techniques of processing big data continue to evolve: what constituted big data 5 years ago is not considered big data now (Crawford et al., 2014); and the nature of what is perceived as ethical is not fixed. Additionally, it is now clearer that accessible or “impersonal” data are not necessarily ethical (i.e., data not classed as personal data are not inherently unproblematic, Zook et al., 2017). Furthermore, even inferential findings can impact human lives in ways which have not been anticipated (Wachter and Mittelstadt, 2019). Furthermore, neuroscience data, beyond their sheer complexity and multimodality, are inherently sensitive and personal in that they possess philosophical relevance with regard to the nature of humanity, and are concomitantly of a personal nature (i.e., derived from an individual and may in some cases be used to identify an individual). These factors amplify the importance of taking responsible approaches to the governance of neuroscience big data by several orders of magnitude. We consider “responsible data governance” to be inclusive of concerns related to the ethical, legal, and social issues which may arise in relation to any use of the data in question, consisting of big data for neuroscience research in this case.

Multiple perspectives on the nature of big data and the potential risks and benefits of related analytics exist, and questions regarding moral, legal, or social responsibilities and issues of privacy and data protection, accountability, and ownership are inherent. Christen et al. (2016) provide an outline of the ethical concerns presented by “Big Neuroscience,” highlighting the fundamental role of big data in this context, and other scholars have discussed ongoing and emergent ethical concerns in big data (Metcalf and Crawford, 2016; Mittelstadt and Floridi, 2016). However, data governance in a more general sense is infrequently linked to ethical issues, with neuroethical approaches lacking direct engagement with data governance (Farah, 2015), and data governance research neglecting ethical aspects (Nielsen, 2017). Additionally, the matter of how responsible governance of neuroscience big data can be operationalised has not yet been addressed, which is one motivation for undertaking the work discussed here. Other factors which have influenced us include internal project needs for sustainability and an approach to data-related ethical, social and legal concerns which forms part of an overall data management strategy. External needs have also been important, especially the swiftly changing regulatory landscape in the wake of the General Data Protection Regulation (GDPR; EU 2016/679) coming into effect and the increasingly global nature of neuroscience research.

This article responds to these needs and will also describe the ongoing development of “responsible big data governance” in the HBP, a large, international collaborative neuroscience project.

In this article, we will address the question of how ethical issues can be integrated into the design and implementation of approaches to neuroscience big data governance by describing and reflecting upon the origin, operationalisation, and continuing development of structures designed for this purpose in the HBP. Our purpose in doing so is threefold: to offer a way of addressing the need for ethical governance of neuroscience big data; to provide an example of this in practice; and to contribute to wider, ongoing conversations around the challenges posed by the responsible use of big data. We provide the intellectual context for this work by reviewing the literature on big data ethics and governance, illustrating the points in the data lifecycle at which issues arise, outlining the approach and methods employed in designing big data governance frameworks using Responsible Research and Innovation (RRI) for the HBP, discuss and reflect upon the outcomes of implementing “responsible data governance,” and conclude with suggested directions for related research.

The article makes several original contributions to the literature: it enriches the big data ethics debate by presenting a novel data governance approach which explicitly incorporates RRI in related structures and processes. Attention to data governance will, in many cases, be a necessary condition for addressing the ethics of big data. Our focus on neuroscience big data demonstrates the close interrelatedness of the ethical issues relevant to big data and broader issues in data ethics. By drawing on our insights from the HBP, we also provide empirical evidence which demonstrates our approach to the governance of neuroscience big data. These insights are likely to be of interest to scholars working on the ethics of neuroscience data or information management, and our conclusions contribute to wider discussions and debates regarding the ethics of big data.

## Ethics and Big Data

Before turning to the HBP, we briefly outline the background and relevant aspects of big data, its characteristics, and the ethical challenges associated with it. These are important for defining the intellectual context of this work and have influenced how the authors define and approach big data.

Big data scholarship has emerged relatively recently; publications dedicated to the topic are legion, and originate from a large, diverse set of contributory disciplines ranging from human geography to information systems. The high number of publications is likely due to the enormous amount of interest and attention drawn to the phrase by the tantalising promise of “data-driven” initiatives enhancing outcomes across vast intellectual and practical terrain, and even famously offering the “end of theory” a decade ago (Anderson, 2008). By 2011, big data and associated analytics were featured ascending the “peak of inflated expectations” in the Gartner hype cycle, with 2 to 5 years until mainstream adoption (Fenn and LeHong, 2011, p. 9). This was accompanied by a flurry of publications, with a critical realist examination 2 years later concluding that this hype dramatically underrepresented the complex interplay of numerous factors which could prevent the technique (or assets) from achieving anything approaching the desired transformations (Fox and Do, 2013). More recently, a single-field meta-analysis characterised big data research as immature and far from unified (Frizzo-Barker et al., 2016). A large, cross-disciplinary synthesis identified a group of issues affecting big data more generally, and these have social and ethical implications: big data are not monolithic; big data are not solely beneficial (see also O’Neil, 2016; Lepri et al., 2017); big data are part of the “regime of futurity”; big data contribute to digital and social divides (Ekbia et al., 2014, pp. 1538–1539).

Furthermore, there is a fundamental question implicit in the use of the term “big data”: Is this a reference to the analytical processes used on large datasets, or does it refer to the actual datasets (e.g., Mittelstadt and Floridi, 2016, p. 309)? Although there is a consensus that big data cannot easily be defined (Crawford et al., 2014; Kitchin, 2014; Mittelstadt and Floridi, 2016), there is also a clear reliance on at least the three characteristics initially defined by Laney (2001), with other traits appended as the concept travelled the course of the hype cycle (Dodge and Kitchin, 2005; Zikopoulos and Eaton, 2011; Boyd and Crawford, 2012; Kitchin, 2013, 2014; Ekbia et al., 2014; IBM, 2014; Marz and Warren, 2015; Mayer-Schonberger and Cukier, 2013; L’Heureux et al., 2017):

- Volume
- Velocity
- Variety
- Veracity
- Value
- Exhaustive, attempting to describe entire systems
- Finely-grained in resolution and indexical in nature
- Relational in nature with common data fields
- Flexible, by way of extensionality and scalability

The much-lauded benefits of big data and associated analytics for operational efficiency, increased revenue, and improved competitive advantages are accompanied by substantial risks. These include privacy concerns, most notably regarding anonymisation and re-identifiability (thus raising serious potential issues of legislative compliance, amongst others), worsening social and economic inequalities through continued contributions to digital divides, and a lack of stakeholder and public trust due to low data quality or the opacity of the analytics. A series of high-profile misuses of big data ranging from the identification of specific individuals from public data to the Facebook and Cambridge Analytica debacles is evidence of the ways in which these risks may routinely be unconsidered or unmitigated. Partly in response to these risks and phenomena, several examinations of the ethics of big data, epistemological shifts, or related future concerns have been published (Davis, 2012; Boyd and Crawford, 2012; Crawford et al., 2014; Kitchin, 2014; Zwitter, 2014; Metcalf and Crawford, 2016; Mittelstadt and Floridi, 2016; O’Leary, 2016; Salerno et al., 2017; Chalcraft, 2018).

Big data governance may thus present an opportunity to practically address ethical concerns and attempt to mitigate some risks whilst preventing the loss of potential social benefits from the work in question. Applying a situated approach to RRI in combination with established data management principles such as the FAIR guidelines (Findable, Accessible, Interoperable, Re-Useable; Wilkinson et al., 2016), would appear to offer a favourable way forward. However, the principal sources on the governance of big data take a business organisation and strategy orientation (e.g., Soares, 2012; Morabito, 2015) and are not fully appropriate to apply to a large, international research project. Moreover, these attempt to combine the monetary profit-driven model of data governance for business with an opaque, vague notion of what big data are, for example: “Big data governance is part of a broader data governance program that formulates policy relating to the optimization, privacy, and monetization of big data by aligning the objectives of multiple functions.” (Soares, 2013, p. 6). Such examples also require top-down implementation and assume homogeneous ethical perceptions and moral positions across organisations.

**Figure 1:**
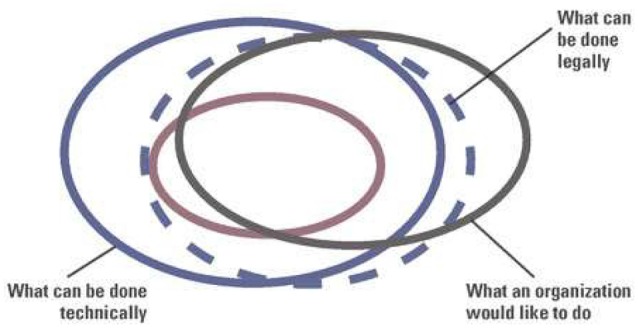
Ethical boundaries of data analytics are shown as a sub-set of the legal boundaries of data analytics (Chalcraft, 2018, p. 19; Figure 1).

In the English-language literature on the ethics of data and its governance, European and North American perspectives dominate the discourse, and the disciplinary scope is limited in comparison to discussions of big data. This imbalance is problematic from the increasingly interdisciplinary, global view being adopted in neuroscience. Even so, essential definitions of concepts such as anonymisation are not shared, the perceived nature of “ethics” or “ethical” varies widely, and thematic tensions and clashes of perspective are apparent. For example, Figure 1 in Chalcraft’s article showing the ethical boundaries for big data analytics depicts all ethical possibilities as the smallest boundary (Figure 1, in red), a sub-set of what can be done legally (from Chessell, 2014; Chalcraft, 2018, p. 19).

Although morality and law may overlap, few ethicists would agree that legality (or regulatory compliance) is equivalent to being ethical. Ethics may also be distilled into tick-box exercises or rulesets, and O’Leary (2016) presents codes of conduct or ethics as the sole manifestation of ethics in computing-related fields. Responsible use of big data is summarised under 10 headings in a practical article by Zook et al. (2017), and although these are wholly relevant, the North American orientation of the piece omits fundamental issues of concern from our perspective (e.g., data subject rights under the GDPR). A conceptual space in which to comprehensively address data ethics, consisting of three axes including the ethics of data, algorithms, and practices has been proposed (Floridi and Taddeo, 2016), but an empirical application of this concept has not yet been offered. Similarly, a recent article offering twenty recommendations for the ethical development of AI, which is fundamentally reliant upon big data, offers no explicit suggestions for supporting ethical data governance despite thorough consideration of related issues (Floridi et al., 2018).

The ethics of open data or open science vs. privacy and data protection, and problems related to data collection, data sharing, anonymisation, and informed consent in human health research are significant, obvious loci for debate in big data research. These discussions often feature conflicting perspectives on human rights concerns, amongst other issues. Sharing data is recognised as a general imperative because it inexpensively improves replicability and transparency, enhances research practice, and can reveal new research questions. Views on the ethics of doing so, however, are not shared and tend to embrace deontological perspectives or consequentialism. In their article on neuroimaging data-sharing, Poldrack and Gorgolewski (2014) argue that data-sharing is an ethical duty of all researchers, to maximise the “contribution of human subjects” (2014, p. 1510). Although they briefly mention that a potential lack of credit or acknowledgment discourages data-sharing, privacy concerns are described as the only relevant “ethical issue,” and these authors propose that subjects simply be provided with more information about data re-use and sharing during the informed consent process (2014, p. 1515). This aligns somewhat with the position advanced by Salerno et al., 2017 who, in a article following a conference of the American College of Epidemiology, state that: “individuals in a health care system have a reciprocal duty not to oppose, and perhaps even facilitate the credible collection and analysis of their data” (2017, p. 300), and frame any human subjects (and, moreover, any approving body) who may object to the collective analysis of their data (or require individual permissions) as barriers to science. As an example of an ethical framework to address big data, they refer to the *Framework for Responsible Sharing of Genomic and Health-Related Data*, which “takes as its starting point the right of all citizens to benefit from advances in science” (Salerno et al., 2017, p. 300). This right, along with fair and equal access to health care systems, is not the default situation, particularly in a US context. Additionally, other issues such as the serious, often localised power imbalances which may prevent this concept from ideal operationalisation, or the future potential for government-approved third parties to use this data, are not addressed.

In other words, human subjects are viewed by some as responsible for donating their data or permitting it to be collected (though this must be balanced against a risk that this might cause harm in the future); researchers are perceived to have a duty to share data (though better mechanisms for allocating credit are needed); and there is engagement required on the issue of informed consent for human subjects. These positions incorporate ethics, but do not consider the full spectrum of ethical issues around big data or the social factors at work; each situation will operate in a different legal and cultural context; and we cannot predict the impacts of big data, particularly regarding group risks. Crawford et al. (2014) describe this problem as follows: “To be concerned about individual risk is equated with hindering progress… …this fails to acknowledge the ways in which our data can reveal much about us that we cannot know or intend, and can be used to discriminate against individuals and groups” (2014, p. 1666). Other authors agree, contending that a core ethical issue with big data is that what can be learned from it in the future is unforeseeable and that the use of big data has an underestimated impact on individuals and groups (Willis et al., 2013; Zwitter, 2014). Furthermore, reflecting upon issues affecting genomics and the expectations of “information altruists,” Choudhury et al. (2014) point out the assumptions of privilege inherent in such expectations, and observe that “only those well-buffered from the social risks of exposing their future health vulnerabilities could afford to volunteer” their data (2014, p. 7). Furthermore, given that some groups will not be represented for a combination of reasons (e.g., refusal, tacit exclusion due to social status or income), a truly representative dataset is unachievable and cannot be obtained through simply getting larger datasets. For an approach to neuroscience big data governance to be successful, these dilemmas and tensions must be acknowledged and addressed inclusively and pragmatically.

### Big Data in the HBP

Defining “big data” precisely is only achievable in a momentary sense since fulfilling even the first criterion, “Volume,” is deeply contextual (Crawford et al., 2014). We, therefore, consider “big data” in the HBP to fulfill at least the criteria of Volume, Velocity, Variety, and Veracity within the descriptive framework used by L’Heureux et al. (2017). In terms of scope, we use “big data” to refer collectively to the datasets and the analytical techniques used to process them because the entire system is of concern when considering governance issues and the points at which ethical issues arise in the data lifecycle.

Volume, the most frequently associated characteristic, does not relate solely to quantities of storage space, but rather to the challenges encountered when using traditional or well-established approaches to process the data in question (L’Heureux et al., 2017, p. 7777). Furthermore, “size” includes dimensionality, and thus may refer to vertical (e.g., the number of samples) or horizontal aspects (e.g., the features a dataset contains) of datasets. Likewise, Velocity refers not only to the speed at which data are created but also to the rate at which data must be analysed in a specific context. Although structural variation in datasets is certainly relevant, both syntactic (data-type diversity) and semantic heterogeneity (interpretational diversity) also fall under the criterion of Variety. Veracity is defined by the quality (e.g., uncertainty, precision, and noise) and provenance (the origin, tracking, and movement) of the data (Gandomi and Haider, 2015; Wang et al., 2015; L’Heureux et al., 2017).

In addition to fulfilling a situated manifestation of Volume, Velocity, Variety, and Veracity, the HBP has been used as an exemplar of “Big Neuroscience” and “big data” science (Wittenburg and Stehouwer, 2015; Christen et al., 2016). Given the increasingly broad spectrum of data-intensive research and the escalation of infrastructure development within the HBP, further criteria may also be met by some parts of the project.

### Ethics and Big Data in the HBP

As a step toward a more comprehensive understanding of the evolving ethical issues relevant to big data and associated analytics, we have summarised these issues in Supplementary Material Appendix S1 (Supplementary Material). These ethical issues, some of which represent conflicting perspectives, must be addressed in a balanced, collaborative, and pragmatic way when designing responsible neuroscience big data governance since the issues will differ by context. In order to fulfill its purposes, a data governance programme should include related dialogues, documents, processes, and workflows as appropriate, and the practical matter of inclusively incorporating future ethical, legal, and social issues into the design and deployment of governance frameworks should also be addressed.

**Figure 2:**
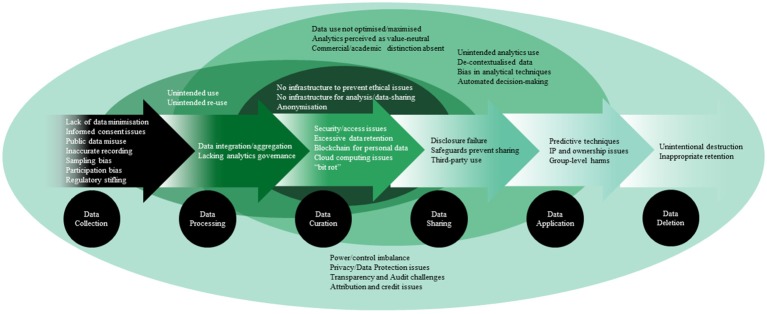
Ethical issues and the overlapping stages at which they may arise in the data lifecycle.

An understanding of ethical issues can be enhanced by considering them within the data lifecycle. Factors which lead to ethical issues are present at different, sometimes multiple, stages, and require different data governance approaches. There is no unified or generally accepted model of data lifecycles, which are contingent upon many factors (Cox and Tam, 2018). Considering the multimodality and disciplinary diversity in neuroscience research, we use a set of overlapping contextual stages (similar to those outlined in Ball, 2012), which can be iterative and recursive but are presented here in a linear format so that the ethical issues are more effectively mapped (Figure 2). We use “data collection” to mean the creation of data and metadata; “data processing” to refer to registration, integration, aggregation, and analysis of data; “data curation” to mean the storage, maintenance, and security of data; “data application” to mean the results of any aspect of data processing; “data sharing” to the provision of access to data; and “data deletion” to refers to the destruction of data. Figure 2 below shows the ethical issues in Supplementary Material Appendix S1 mapped onto a linear view of a data lifecycle. This depiction is not comprehensive but is intended to illustrate the scope and complexity of issues that are relevant to big data and associated analytics.

The inherent issues of complexity, definitional diversity, conflicting views on essential concepts (both within and outside of neuroscience and ICT), human rights being used to justify disparate positions, and a lack of practical application of theory have contributed to our motivations for developing “responsible data governance” for neuroscience big data. Our case study, the HBP, is large, complex, collaborative, and international. It, therefore, possesses attributes requiring special consideration when incorporating ethical frameworks, including a need for a non-imperialist, dialogical approach; cross-disciplinary utility; and sensitivity to social and cultural issues at multiple levels (Aicardi et al., 2018b; Stahl et al., 2018, 2019). In applying RRI within the HBP, the approach to ethical issues has been characterised by dialogues, flexible, and responsive to project needs (Stahl et al., 2016; Aicardi et al., 2018a). We view the creation and continuing development of “responsible data governance” to be part of these efforts.

## Responsible Data Governance, Data Governance Meets RRI

To avoid reducing applied ethical frameworks to tick-box exercises or imposing an ethically-imperialist methodology on a vast collaborative project comprised of diverse cultural and interdisciplinary perspectives, our approach to data governance of neuroscience big data is aligned with that of the Ethics Support work package in the HBP and operationalises an ethics of dialogue based on Habermasian discourse ethics (Habermas, 1991, 2006; Stahl et al., 2019). This approach was designed for responsive, discursive inclusivity in the context of neuro-ICT innovation, with the potential emergence of complex and novel ethical issues in mind. It represents an attempt to find pragmatic solutions rather than adopting a single, fixed moral position, and working across ethical tensions (such as those outlined in section Ethics and Big Data) through ongoing dialogues with internal and external stakeholders. Utilising an ethics of discourse is essential for “big data” governance in the HBP since an understanding of the context and qualities of datasets is necessary for success (the issues raised by clinical neuroimaging data are distinct from those raised by theoretical neuroscience data or data used for neurorobotic simulation). For the purpose of ongoing data governance in the HBP, this means maintaining these dialogues and accepting the existence and importance of competing value structures and multiple moral positions on the governance of neuroscience big data whilst supporting compliance measures.

We therefore introduce “responsible data governance” as an approach to neuroscience big data that explicitly recognises the ethical issues raised throughout the data lifecycle, and aims to address them in a way that acknowledges the fluidity and plurality of definitions, perspectives, and contexts across a large neuroscience project and avoids the pitfalls of ethical imperialism (Stahl et al., 2019). This form of data governance builds upon more general data governance approaches by supplementing our applied ethics of dialogue with ideas from RRI.

### Data Governance

Data governance is conceived of and described in different ways depending upon the institution and discipline in question, but a common, widely accepted definition of the term is not consistently recognised. Many definitions of data governance have been influenced by ICT governance and relate to the use of a framework for accountability and decision-making rights regarding all aspects of data as part of ICT, often intended for a business rather than a research environment (e.g., Weill and Ross, 2004; Weber et al., 2009). Data governance is partly perceived as falling within the domain of regulation, with some praise for responsive legislation such as the GDPR (Chalcraft, 2018), acknowledgment of the interjurisdictional issues that data-sharing presents (Salerno et al., 2017), and other scholars raising concerns that overly restrictive legislation designed to prevent harm will also prevent positive outcomes (Mittelstadt and Floridi, 2016).

Likewise, the purposes of data governance are interpreted in a variety of ways, although coordinated action on data governance is widely perceived as essential to the success of any enterprise, regardless of its nature. According to Nielsen, many goals of data governance may exist (e.g., strategic management of data as an asset, encouraging desirable behaviour related to data, assisting data security), but the primary goal of data governance across practical and disciplinary boundaries is often to maintain data quality, mainly in line with “business goals” (2016, p. 121). Brous et al. (2016) lay out the main principles of data governance as: organisation, alignment, compliance, and common understanding. Even these may be difficult to extend in a general sense due to essential challenges in the presentation and operationalisation of the concept.

Perhaps in part due to the inchoate nature of modern data governance research, the literature is patchy, fragmentary and some fundamental issues are apparent. For example, the terms “information” and “data” are often used interchangeably, and a distinction between information governance and data governance may not be evident (Nielsen, 2017). In a defining volume dedicated to data governance, data are framed as an asset for management, a source of economic value, or a resource for extraction (Ladley, 2012), which reflects a somewhat narrow view of what data are or could be, and a broad lack of engagement with data-related social and ethical issues. Even Nielsen’s purportedly comprehensive review of related literature fails to represent the complete picture of data governance discourse by electing only to include computer science, information systems, e-health, management and organisation, education, and e-government fields of practice (Nielsen, 2017, p. 127). This excludes a range of fields in which data governance is fundamental to success, including finance, marketing, and engineering. Another issue is the question of who is responsible for the design and implementation of data governance, with some authors calling for a move away from ICT-controlled data governance strategies and toward groups with multiple specialisms or disciplines represented (Weber et al., 2009). Apart from the significant issues of framing, definition, and responsibility, data governance faces substantial technical challenges of implementation, which are contextually-contingent. Although ethics have surely played a role in the development of many conventional systems of data governance, a full consideration of these relationships is still lacking.

We define data governance as a strategy for the overall management of the availability, usability, integrity, quality, and security of data in order to ensure that the potential of the data is maximised whilst regulatory and ethical compliance is achieved within a specific organisational context. To address data governance within the HBP, we have operationalised the described theoretical approach and incorporated the principles of RRI into the data governance policies, processes, and infrastructure of the project through the establishment of dialogues and shared responsibility (Stahl et al., 2018, 2019).

### Responsible Research and Innovation

RRI is an approach to the governance of research and innovation which aims to unite and respond to the main stakeholders and their expectations. Building on a long history of research and innovation governance that includes technology assessment (Grunwald, 2011), philosophy of technology, science and technology studies, and others, RRI has gained prominence from around 2010. One of the most widely cited definitions of RRI states that it aims to ensure the acceptability, desirability, and sustainability of research and innovation processes as well as their outcomes (Von Schomberg, 2011). The idea of RRI was embraced by some national and international research funders, arguably because they saw value in ensuring that tax-funded research benefits the taxpayer. Although other approaches to research and innovation governance have been employed in different regions, the highest-profile adopter of RRI was the European Commission (European Commission, 2013).

One prominent version of RRI is based on Stilgoe et al. (2013), which was modified and adopted by the UK Engineering and Physical Sciences Research Council (Owen, 2014). In this view, RRI is characterised by four separate but interlinking features: Anticipation, Reflection, Engagement and responsiveness (or Action, thus the AREA framework). This means that research and innovation activities need to consider possible future consequences, have integrated mechanisms of ensuring and fostering reflexivity, must reach out to relevant stakeholders and engage with them, and be willing and able to respond to the stakeholders and act accordingly. These characteristics increase the appeal of using RRI in large, complex research projects, as opposed to more rigid approaches like technology assessment.

These activities and interactions are clearly aimed at bringing together the various stakeholder groups within the ecosystem of research and innovation to ensure an ongoing, productive dialogue that allows research and innovation to be steered in ways that participants in the dialogue agree are beneficial.

This is a highly ambitious aspiration, and it raises several fundamental and practical questions. For example, it is not clear whether heterogenous societies can reach a consensus on what counts as desirable or acceptable. The question of who counts as a stakeholder and who is to be legitimately included in debates is difficult to answer. The translation of a consensus into practical action is also not a trivial matter. In addition, these activities require resources which need to be diverted from other objectives. The space to comprehensively discuss these questions is not available here, but there is agreement that RRI has the potential to address some of the most pressing issues surrounding research and innovation and the governance thereof. This is true across most disciplines, but there has been specific recognition of the importance of RRI in ICT (Jirotka et al., 2017).

### Combining Data Governance and RRI: Responsible Data Governance

“Responsible data governance” therefore consists of activities that integrate the principles of RRI into data governance structures and processes by applying an ethics of dialogue. This means that data governance should be structured in a way that facilitates reflexivity, integrates foresight and consideration for future impacts, is open to stakeholder engagement, and is flexible enough to be modified according to the outcomes of these activities and the discourses surrounding them. In addition, “responsible data governance” must build on the good practices established in traditional data governance to ensure that the technical infrastructure is sufficient, sound, and secure, and that all processes and workflows involving data are developed and maintained at high standards.

To avoid any potential misunderstanding, we wish to clarify that by introducing the term “responsible data governance,” we do not intend to imply that any other data governance approach is irresponsible. Good data governance that does not incorporate the term “responsible” may well be data governance that is responsible, and in many cases will be an appropriate way of addressing similar data-related issues.

We think that “responsible data governance” as described here is particularly necessary in certain circumstances, particularly those in which a high degree of complexity exists in multiple dimensions, e.g., in terms of users and stakeholders, data types and quantities, users of data and technologies employed for data processing and analysis. In such cases, it will be difficult if not impossible for any individual or any single scientific discipline to fully comprehend the potential implications and consequences of certain choices as they relate to data governance. The uncertainty created by complexity extends to the technical side of data governance, but we focus here on questions of ethical and social relevance. Framed differently, “responsible data governance” is called for in those cases where the ethical implications of the collection, processing, use and sharing of data are difficult to foresee but likely to be of significant importance. This is certainly the case with neuroscience big data, where data governance is likely to benefit from structures that expose discussion to scrutiny, that facilitate involvement of a variety of voices, and that incorporate incentives for creators and users of data to reach out to the other stakeholders who are likely to be affected by their work. Table 1 demonstrates the implementation of “responsible data governance” through general examples of actions taken at different data lifecycle stages within the AREA RRI framework.

**Table 1:** Examples of “responsible data governance” actions and relationships over the data lifecycle, arranged within the AREA RRI framework.

	Anticipate	Reflect	Engage	Act
Data collection	Potential social and economic impacts, data minimisation, consent	On interest in and need for research, value	Inclusive dialogue with communities, data subjects, potential users	Create guidelines and regulation, ensure these do not stifle innovation
Data Processing	Future interoperability and registration issues and impacts	On reasons for types of processing, which metadata retained	Dialogue with those registering data, analystics	Formalise and regularly update data handling practices
Data curation	Potential access, security, degradation impacts, changes to consent, user or data subject requests and impacts	Duration and justifications for curation, changing security measures	Dialogue with users, platform or database admins	Maintain secure infrastructure, technical oversight, breach/reporting practices in place
Data sharing	Possible user, data subject social and economic impacts	Users motivations and purposes, credit for data, implications of re-use	Dialogue with users, groups or community stakeholders	Guide, regulate, and modify responsible access
Data application	Social and economic impacts to user, data subject	Varied uses or de-contextualised interpretations	Dialogue with subject experts, users, groups or community stakeholders	Report and publish even negative results, monitor outcomes
Data deletion	Potential consequences of removal or retention of data	Backups or copies of data, metadata conflicts or errors	Dialogue with users, platform and database admins	Responsively develop data retention schedules and workflows to inform collection stage

In the following section, we outline the methodology used to develop “responsible data governance” and apply it within the context of a complex, multidisciplinary, big neuroscience research project.

## Materials and Methods

The “responsible data governance” approach in this article has been developed in an iterative way, combining deductive concepts (derived from literature) with inductive approaches (empirical insights from an in-depth case study; George and Bennett, 2005). Continuously comparing existing concepts with empirical insights and refining the emerging approach accordingly increases the validity of the approach. The development of an approach “occurs *via* recursive cycling” with respect to the case data, the approach, and the relevant literature (Eisenhardt and Graebner, 2007; p. 25). A case study is a particularly useful method for developing an accurate, relevant, and interesting approach because it permits the study of complex phenomena in depth, providing detailed and nuanced understandings and contributing rich insights from a real-world context (Eisenhardt, 1989; George and Bennett, 2005; Eisenhardt and Graebner, 2007; Yin, 2014). We have followed these principles in constructing strategies for neuroscience big data governance through a similar developmental process and have undertaken a detailed case study of the HBP alongside our research into data governance, which has shaped each other in a cyclical manner. The HBP provides a data-rich and historically important case within which to consider data governance because it is one of the largest international scientific projects to date, with more than 100 institutional partners in more than 20 countries. Furthermore, the overarching project aim of infrastructural development to support future neuroscientific research (Amunts et al., 2016) contributes to our ongoing conceptual insights on data governance.

HBP-related insights originate from multiple data sources, including analysis of legislation, internal documents, and observations of project meetings. All authors of this article are researchers involved in developing and implementing “responsible data governance” in the HBP, which has provided us with first-hand experience and access to extensive internal information. Whilst the combination of scientific research, practice, and regular involvement in external reviews facilitates reflection and awareness of the complexities and challenges involved in data governance, our status as members of the project can also lead to a degree of self-censorship and may open up the possibility of actively or unconsciously withholding information that may not reflect well upon or be viewed favourably by the project.

The HBP offers three important applications for developing the “responsible data governance” approach: motivation, inspiration, and illustration (Siggelkow, 2007). First, it provides a motivation for asking how responsible governance of big neuroscience can be operationalised. Rather than having a purely theoretical motivation, the HBP case presents an urgent real-life necessity to operationalise a responsible data governance approach. Second, immersion in the intellectual environment of the HBP provides inspiration for new ideas to advance the “responsible data governance” approach in an inductive way, and thus also serves to complement and refine deductive insights from the literature. Last, the HBP vividly illustrates the conceptual arguments and empirical challenges facing responsible governance of neuroscience big data.

## Results

### Ethics and Neuroscience Big Data Governance in the Human Brain Project

Ethics Support is a work package within the Ethics and Society subproject of the HBP that includes data governance-related tasks such as compliance, ethics-related data governance, and data protection. These tasks are part of the greater HBP ethics support infrastructure, designed to address the ethical and social issues that arise in the project (Stahl et al., 2016, 2018). Here we will detail some ethical issues raised by the handling of neuroscience big data within the HBP, and the measures implemented to address them.

The HBP presents data governance challenges due to the scientific complexity of its subjects and the diversity of data modalities data handled. Groups in over 100 universities, hospitals, and research centres across Europe contribute data to the HBP, and the main contribution from many HBP research partners into the intended research infrastructure will be big data. Development of data governance structures and processes within the HBP has been driven by internal project needs as well as external processes such as the need to ensure compliance with the GDPR. In 2017, the Ethics and Society subproject published the *Data Protection and Privacy Opinion and Action Plan* specifying the relevant issues and concerns in this area, as well as the contingent ethical considerations within the HBP (HBP, 2017). This led to the creation of a Data Governance Working Group (DGWG, a group of cross-project representatives and experts responsible for creating data governance policy) and the appointment of a Data Protection Officer (DPO). In addition to these, the Researcher Awareness task in Ethics and Society was set up to support researchers in reflection on related potential ethical and social issues. Additionally, individuals in each subproject called Ethics Rapporteurs establish and maintain ethics-related feedback links throughout the HBP and are critical to continuing ethics-related dialogues, raising awareness of data governance issues, and creating related policy. Independent structures have been created to clarify complex situations, including the Ethics Advisory Board and an Ombudsperson. To raise wider awareness of data governance issues with internal and external stakeholders, the HBP has organized dedicated workshops and educational activities. Other, more technically-oriented HBP groups are also involved in the data lifecycle, and the foremost of these is the Data Curation team, whose role is explained in more detail below.

The HBP has set up technical platforms that will be jointly operated to allow for collaboration and data sharing across Europe as a fundamental infrastructure for neuroscience innovation. In order to achieve this, data must travel from its source: in a lab, hospital or other research institution, to the platform from which it can be shared with the interested research community across the continent or elsewhere. This is no simple task, however, as when data crosses jurisdictional boundaries (e.g., between institutions, Member States, or across larger-scale boundaries such as those around the EU), we must consider the ethical and legal implications of this action. Thorny issues such as where (and with whom) responsibility for that data now lies, what laws govern it, where and in what state might it be registered, stored, shared, or destroyed and what rights the original data subjects have over its use must be considered. These issues are magnified when the data being brought into HBP platforms has been produced in a country which falls outside the GDPR (i.e., USA, Asia, South America, etc.). These are locations where vast amounts of neuroscience big data are being produced (which may have a high potential value in terms of scientific advancement), but how can the HBP ensure that the data they accept into their platforms adhere to acceptable ethical standards? Tackling such issues is paramount to conducting responsible research in an environment built upon collaborations across complex jurisdictional boundaries.

In terms of ethical compliance, big data present several challenges to the HBP. One such challenge is how the attribution of responsibilities changes throughout the data lifecycle. In order to show how responsibility for compliance is managed in the HBP, we will briefly outline the ethical compliance processes in the project.

In broad terms, there are two events in the data lifecycle where the official ethical compliance processes instantiated within the HBP interact with a project aspect utilising big data. The first is in the early stages of a research projects’ integration into the HBP, when the internal ethics checks ensure that data type experts within HBP Ethics Support review all appropriate regulatory documentation. At this point, the HBP DPO may raise any concerns concerning the data handling features of the incoming project. Any issues are resolved through dialogue between the DPO, the research team and other Ethics Support members. One outcome of this dialogue (and the ultimate completion of the ethics check) is a record in the Ethics Registry, a database of all HBP tasks and their compliance status. The Ethics Registry is kept in Tresorit, an end-to-end encrypted cloud storage service, and is simultaneously a reporting tool that allows Ethics Support (and European Commission reviewers) to quickly access the ethics compliance status of all tasks stored within the database, and a live document used by HBP members to track and record ongoing compliance checks for all tasks. In keeping with an ethics of dialogue, the exchanges and relationships necessary to maintain the Ethics Registry are accomplished through ongoing, inclusive discourse.

Ethics Support is explicitly not an ethics committee and cannot give ethical approval for studies within the HBP. Instead, that responsibility belongs to the local regulatory authorities across the EU and institutional ethics approval boards where the research is to take place. The ethics compliance-related responsibility of Ethics Support is to ensure that the appropriate documentation is in place before the project is integrated into the HBP and that this submitted documentation is appropriate to account for the activity in the project. The second compliance-related interaction comes later in the lifecycle when the data produced by the project is uploaded into the HBP platforms for storage (in the future, the intended public-access repository is the KnowledgeGraph). At this point, the Data Curation Team in subproject 5 (the Neuroinformatics Platform) conducts an ex-post check to ensure that all the appropriate compliance information is present for the project that produced the data. In practice, this process involves a crosscheck between the compliance information supplied by the data provider, and the compliance documentation recorded by HBP Ethics Support in their Ethics Registry. At this stage, if there are any concerns, issues or any incongruence between the two sources of information (data provider and ethics registry) then this is resolved through dialogue and collaboration between Ethics Support, the Neuroinformatics Platform Data Curation team and the data provider. These two events, the ex-ante checks conducted by Ethics Support and the ex-post checks conducted by the Data Curation team are, on the surface, neutral acts of administration—the processes check that all documentation is in place and record the outcomes. In reality, these processes are reliant on dialogues and reflect the ethics of practices (Floridi and Taddeo, 2016). They require ongoing discourse across HBP internal research ethics experts, the scientists in the relevant field, and the various governance structures both within the project and at the European Commission to ensure that they are resolved appropriately. Furthermore, the experiences of these dialogues feed into the formation of data governance policy.

### The Medical Informatics Platform

The human subject data produced in subproject 8: The Medical Informatics Platform (MIP) serves as an operational example of these processes and structures working together. The MIP is a collaborative, open-source platform that will allow researchers to share anonymised medical data worldwide. Most of the research data produced by SP8 are collected from human participants, meet the criteria for big data outlined in section 2.1 (L’Heureux et al., 2017), and are both personal and sensitive. These data, therefore, pose several challenges for ethical compliance and data governance.

In some HBP projects, the standard compliance process might be as simple as a discussion regarding the nature of the activity. Numerous tasks in the HBP involve work on platform development, for example, pose very few (if any) ethical issues, do not warrant any ethical approval, and no formal check for compliance is applicable. In such cases, discourse on the future impact of this work is used to foster ethical reflection. In the case of research related to the MIP however, it is likely that the data are of a clinical nature and that the research involves human participants, perhaps in the care of a hospital, and a closer examination coupled with more in-depth discussion are therefore necessary. As part of this, participant information sheets and consent forms are collected, as well as information regarding sensitive data handling measures that the study team has implemented. These documents are then reviewed by the appropriate internal research ethics experts—e.g., a human research ethics expert for issues of consent and related medical matters, and the DPO for the handling of personal data. The internal research ethics experts work closely with Ethics Support staff, including the compliance manager, Ethics Director and other ethics experts within the team, the subproject 8 Ethics Rapporteurs, and researchers to resolve any issues which might arise from a review. Recurrent questions in medical research include how to handle incidental findings emerging from the research procedures, and who may provide consent for those who are unable to actively consent. Blanket answers to such complex questions would be administratively convenient. However, it would be out of step with the dialogical approach of Ethics Support and responsible data governance (and entirely impractical) to forego opportunities for discursive ethical reflection and fail to take local research contexts into account. The outcome of each series of dialogues is intended to be situationally-specific.

Furthermore, HBP researchers in subproject 8 are expected to follow standard HBP guidelines regarding data handling and data protection. Should any queries or complications regarding data governance or data protection arise, several sources of expert advice and avenues to raise concerns or queries are available. The study team can contact their Ethics Rapporteur or the Ethics Support team, where they can seek expert ethical advice, including from the DPO. MIP researchers may also contact their subproject representative on the Data Governance Working Group for advice at a project-wide level. As with the general ethical compliance processes, such dialogues are key to ensuring reflection on ethical issues, supporting an awareness of data governance, and contributing to the ongoing development of the approach.

Once a dataset is collected, it must be fully anonymised before being made available on the MIP for utilisation in further research. Questions of responsibility become more complex at this stage as the data are made available worldwide and are further removed from researchers. Therefore, it is critical that this process is managed appropriately and that the data are handled in line with relevant legislation and internal HBP policy. The Data Governance Working Group is pivotal at this point in the process as they develop HBP policy informing data management and represent a resource of interdisciplinary project-wide expertise. Additionally, once the medical data are anonymised, curated and uploaded to the MIP, the connection that data has to its original subject is immensely complexified—so the ongoing outreach to participants of HBP research is very important and presents another example of why communication and dialogue are essential in all aspects of the process.

The MIP data have undergone modal changes, collected in the first instance from participants and ultimately intended for the use of a community of researchers worldwide. These big data are transitioning from local to global, personal to anonymised and from individual participant to massive cohorts. Elements of this process are managed administratively, and boxes must be checked to ensure adherence to legislation, but the dialogical interface between numerous stakeholders within and outside of the project ensures that this process is handled inclusively and in a contextually-appropriate way.

### Responsibilities and Challenges

The HBP has a responsibility to ensure that all projects under its banner are fully compliant with appropriate EU regulations. In theory, should the above-described processes be thoroughly conducted, it can be assured that this is the case. In practice, however, there are complexities in these processes that emerge when dealing with neuroscience big data. Due to the transitory nature of many datasets, having been moved from a research institution, to a hospital, to a university and so forth, sometimes repeatedly or over an extended period, it can be difficult to collate compliance information relating to the original data collection context, particularly if metadata has been lost. This is made doubly complex when many datasets are publicly available online and are so widely used that their ethical compliance is assumed and not made explicit to the scientist—another potential example of the problematic nature of public data (Zook et al., 2017). For big data gathered outside the EU, how can the HBP assess the equivalency of compliance documentation produced in countries that fall outside the jurisdiction of EU data protection guidelines? The HBP internal ethics compliance processes rely upon the logic that a local authority approval provided to a study in one EU country is equivalent in worth and function to one produced in a different EU country. When reviewing ethics documentation from outside the EU, this equivalency cannot necessarily be assured. Indeed, non-EU countries with perfectly defensible ethical positions may nonetheless not adhere to EU legislation and guidelines. When data are collected in more than 100 individual institutions across Europe, how can the HBP reconcile the variance in compliance processes in those local authorities and come to a unified decision about their appropriateness to each study? What responsibility does the HBP have to ensure that once big data are uploaded to an HBP platform, any further research activity to which they are applied is conducted ethically? These are fundamental questions regarding the attribution of responsibility in big data neuroinformatics, and although further discussion of these issues is merited, they fall outside the scope of this article.

Concerted efforts are underway in the HBP to address data-related ethical issues through different frameworks, groups, roles, and relationships which all contribute to the deployment of “responsible data governance” in the project. Table 2 shows some examples of the structures, roles, and relationships involved in actions to support “responsible data governance” in the HBP throughout the data lifecycle.

**Table 2:** Examples of HBP-specific structures, roles, dialogues, and relationships supporting “responsible data governance” throughout the data lifecycle, arranged by the AREA RRI framework.

	Anticipate	Reflect	Engage	Act
Data collection	Local regulatory bodies, HBP researcher awareness dialogues with PIs, researchers	Research PIs and researchers on impact of collection, comply with local ethics approval boards/processes	Open societal and wider stakeholder engagement	Research PIs, researchers, Ethics Support dialogues inform researcher awareness, HBP Data Policy Manual
Data processing	Compliance/internal ethics check, DPO and legal basis for processing	Tri-lateral meetings w/Ethics Advisory Board, Compliance, Rapporteurs	DPO/Ethics Support dialogues, DGWG policy available online	Ethics Registry record made, dialogues with data type experts, transparency efforts
Data curation	DGWG and Data Curation team planning, ensure auditability	Ethics rapporteurs, guidance and policy from DGWG	Data Curation Team, Ethics Support, Research PIs/data provider dialogue	Ethics Registry matching, DPO, processes altered by feedback in dialogue, HBP Data Management Plan
Data sharing	Data Curation team on technical aspects, interoperability, DGWG, DPO on policy, ethical, legal issues	Data Curation Team, DGWG and other policy/governance bodies on implications, metadata and credit	Data Curation team dialogues with Research PIs, Ethics Support	Data Curation team and DGWG responsively update workflows, policy
Data application	Researcher awareness, Researchers/Rapporteurs consider implications of data use	Researchers, Rapporteurs, Ethics Advisory Board, Ombudsperson if external expertise needed	Research PI, Data Curation team dialogues, attribution appropriately assigned	KnowledgeGraph for anonymised, retained outcomes/results
Data deletion	DGWG, DPO, Data Curation team balance legal, social, ethical aspects	Research PIs, Rapporteurs, Data Curation team consider implications of deletion vs. retention	Research PIs dialogue with Data Curation Team/DPO	Data Curation team destroys or retains, records actions to inform future iterations

These examples are not comprehensive; although conversations and relationships with other internal groups and stakeholders are perhaps more fluid and situational (e.g., platform coordination bodies, subproject Managers, HBP governance bodies), they also contribute to “responsible data governance” in practice.

## Discussion

Applying the “responsible data governance” approach to neuroscience big data has achieved positive outcomes which are unlikely to have occurred in a formal bureaucratic system designed solely for compliance. As exemplified by the HBP generally and the case of data in the MIP in particular, taking this approach has facilitated interdisciplinary and cross-cultural knowledge exchange, supported the preparation of sustainable dialogues and structures to address future ethical issues, and permitted us to develop creative, responsive policy through the participation of project-wide representative bodies.

The development of this approach is an ongoing process, and we aspire to continuously align it with the cyclical model of RRI using the AREA framework referred to earlier, where the final Act is intended to inform the initial Anticipation step in the iteration to follow. The use of dialogues at every step of the data lifecycle is in keeping with the overall approach to ethics in the HBP, which incorporates meta-responsibility and has been designed to avoid some of the pitfalls of ethical imperialism (Stahl et al., 2019). Even so, developing an approach to data governance suitable for big neuroscience has not been without challenges. Within the multi-scalar context of the HBP, practical and social issues abound and range from the sheer expense of secure data storage to the struggle to reach a cross-project consensus on a single aspect of data policy. Thus, there is a risk that any desire to generate truly innovative solutions, new interpretations, or a novel compromise may be quashed by short timelines and competing priorities.

One of the fundamental purposes of data governance is to maintain data quality and integrity so that its use can be maximised, regardless of whether it is viewed solely as a protected asset, an extractive resource, or otherwise (Ladley, 2012). By developing the “responsible data governance” approach for neuroscience big data and incorporating the principles of RRI within it, we support the argument that an essential feature of “high quality data” is how ethical it is. This assertion is made in light of current scholarly understandings of the ways in which big data fundamentally alter the nature of moral responsibility and shift our epistemological understandings (Crawford et al., 2014; Kitchin, 2014; Mittelstadt and Floridi, 2016). It also aligns to some extent with Floridi and Taddeo’s macro-ethical vision of data ethics (2016), and we are in the position to offer a view from the standpoint of data governance practitioners. However, as we have mentioned, there are myriad perspectives on what makes data ethical.

The wide range of views on these issues is why the dialogues that characterise our approach to responsible governance of neuroscience big data in the HBP are necessary and inform the continuous re-shaping of the process itself. The perspectives of neuroscience, social science, and ICT practitioners are essential to the success of “responsible data governance,” just as they are critical to the implementation of RRI. Values vary across countries, disciplines, institutions, and individuals; therefore, ethics cannot simply be outsourced or assigned to one group; the approach we offer here is effective because it strives to incorporate a representative balance of views.

## Conclusion

In answering the question of how ethical issues can be integrated into approaches to neuroscience big data governance, we have contextualised and presented the development of “responsible data governance” to address the need for ethical governance of neuroscience big data in the HBP.

The essential aspects of “responsible data governance” in this respect are an inclusive, discursive ethical approach and the incorporation of the tenets of RRI within collaboratively-developed parameters and frameworks, which are modified in response to ongoing dialogues, changes in legislation, and other features of the technical and regulatory landscape in which the HBP operates. We have illustrated some points at which ethical issues may arise in the data lifecycle and provided examples of how our approach is practically applied in order to contribute to ongoing discussions and debates around the ethics, uses, and futures of big data and associated analytics.

Our approach will continue to develop over time in response to the outcomes and concerns raised within the dialogues that structure it, as well as changing project needs and external factors. Responsible governance of neuroscience big data fundamentally relies upon discourse to successfully incorporate RRI, addresses legislative and ethical compliance requirements, and considers the entire data lifecycle.

Future data governance research should more fully address the integration of the technical aspects of “responsible data governance,” including data security and cyber security issues; investigate potential avenues for establishing international “ethical data” standards; and consider how the “responsible data governance” framework could be applied in other disciplines, and at different scales. With regard to the second point, we strongly recommend the establishment of an international data governance working group dedicated to the development of policy to support responsible governance of big data neuroscience in the context of global collaboration. Such a group should be comprised of data type and technical experts, applied ethics practitioners, representatives from the IBI, IBRO (International Brain Research Organization), INCF (International Neuroinformatics Coordinating Facility), patient advocacy groups, and other stakeholders.

## Author Contributions

BF is responsible for the conceptual framework, figures, tables, and overall narrative of the article, with written content from WK, BS, and IU. All authors contributed to the text and references.

## Conflict of Interest Statement

The authors of this article conducted this research whilst employed by the Human Brain Project, which was used as the case study described herein.

## Supplementary Material

DATA SHEET S1 Ethical issues, associated data lifecycle stages and references.
