# Supplementary material for: Responsible Data Governance of Neuroscience Big Data
Source: Front Neuroinform. 2019 Apr 24;13:28. doi: 10.3389/fninf.2019.00028 (PMC6499198; doi:10.3389/fninf.2019.00028)
Supplement: DATA SHEET S1 — Ethical issues, associated data lifecycle stages and references. [file Data_Sheet_1.docx]

Appendix 1

| **Issues with ethical implications** | **Data Lifecycle Context** | **Reference(s) or example** |
| --- | --- | --- |
| Complexity/scale of big data/analytics prevents transparency/auditability | All phases | Mittelstadt and Floridi 2016; Zook et al. 2017 |
| Privacy/data protection protocols not used | All phases | Christen et al. 2016; Mittelstadt and Floridi 2016; Salerno et al 2017; Zwitter 2014 |
| Power/control imbalance, i.e. over personal data/subject participation not possible/inadequate or in large-scale analysis | All phases | Chalcraft 2018; Choudhury et al. 2014; Metcalf and Crawford 2016; Mittelstadt and Floridi 2016; Salerno et al. 2017; Willis et al 2013; Zwitter 2014 |
| Attribution/credit/authorship protocols not followed | All phases | Choudhury et al. 2014; Salerno et al. 2017; Poldrack and Gorgolewski 2014 |
| Data minimisation not followed | Data collection | Chalcraft 2018; Zook et al 2017; Zwitter 2014 |
| Informed consent incomplete/missing | Data collection | Chalcraft 2018; Christen et al. 2016; Choudhury et al. 2014; Mittelstadt and Floridi 2016; Zook et al. 2017; Zwitter 2014 |
| Unintended use of public data | Data collection | boyd and Crawford 2012; Chalcraft 2018; Choudhury et al. 2014; Metcalf and Crawford 2016; Zook et al. 2017; Zwitter 2014 |
| Inaccurate data recording/metadata | Data collection | Sivarajah et al., 2017; Poldrack and Gorgolewski 2014 |
| Sampling bias | Data collection | Crawford et al. 2014; Kitchin 2014; Mittelstadt and Floridi 2016 |
| Data subjects/patients choose not to participate | Data collection | Mittelstadt and Floridi 2016; Salerno et al. 2017 |
| IRB/ERB or inappropriate regulatory framework stifles research | Data collection | Salerno et al. 2017 |
| Failure to accurately integrate/aggregate data | Data processing | Mittelstadt and Floridi 2016; Sivarajah et al., 2017; Zook et al. 2017 |
| Inadequate oversight/governance/regulation for BD analytics | Data processing | Metcalf and Crawford 2016; Zook et al. 2017 |
| Use of data for unintended/undisclosed purpose | Data processing/collection | Chalcraft 2018; Zwitter 2014 |
| Re-use of data for unintended purpose | Data processing/collection | Chalcraft 2018; Metcalf and Crawford 2016 |
| Personal data insufficiently anonymised or re-identified | Data processing/curation | Mittelstadt and Floridi 2016; Willis et al. 2013; Zook et al. 2017 |
| Inadequate policy or data infrastructure to prevent ethical issues | Data processing/curation | Chalcraft 2018; Christen et al. 2016; Mittelstadt and Floridi 2016; O'Leary 2016 |
| Inadequate policy or data infrastructure to support data analytics/sharing "Big Data Divides" | Data processing/curation | boyd and Crawford 2012; Crawford et al. 2014; Mittelstadt and Floridi 2016; Poldrack and Gorgolewski 2014; Salerno et al. 2017; |
| Bias/discrimination in analytical techniques | Data processing/application | Chalcraft 2018; Kitchin 2014; Zook et al. 2017 |
| Decision-making by automated techniques | Data processing/application | Chalcraft 2018; Floridi and Taddeo 2016; Willis 2013 |
| Use of analytics for unintended purpose | Data processing/application | Chalcraft 2018 |
| Data not contextualised/informed by epistemology/theory/social aspects | Data processing/application | boyd and Crawford 2012; Crawford et al. 2014; Fuchs 2017; Kitchin 2014; Mittelstadt and Floridi 2016; Zook et al. 2017 |
| Data use not optimised/maximised | Data processing/application | Christen et al. 2016; Salerno et al 2017 |
| Big data perceived as value-neutral | Data processing/application | boyd and Crawford 2012; Crawford et al. 2014; Mittelstadt and Floridi 2016; Willis et al. 2013; Zook et al. 2017; Zwitter 2014 |
| No distinction between use of big data for commercial/academic research | Data processing/application | Mittelstadt and Floridi 2016 |
| Retaining data for longer than necessary/failure to destroy data | Data curation | Chalcraft 2018; Mittelstadt and Floridi 2016 |
| Security of data incomplete/inappropriate access; Data breach/unauthorised disclosure | Data curation | Chalcraft 2018; Crawford et al. 2014; Salerno et al. 2017; Zook et al. 2017 |
| Use of cloud computing for personal data | Data curation | Risk of sharing data with third parties by providers as part of "data for service" |
| Use of blockchain for personal data (esp EU context) | Data curation | Violation of data subject rights under the GDPR |
| Degradation of digital information/"bit rot" | Data curation | Haslop et al. 2017 |
| Failure to make data public | Data sharing | Choudhury et al. 2014; Poldrack and Gorgolewski 2014; Salerno et al. 2017 |
| Data-sharing inappropriate/third party use | Data sharing | Chalcraft 2018; Zook et al. 2017 |
| Data safeguards prevent data sharing | Data sharing | Mittelstadt and Floridi 2016; Poldrack and Gorgolewski 2014; Salerno et al. 2017 |
| Unwarranted prediction by automated techniques | Data application | Sivarajah et al., 2017; Willis et al. 2013; Zwitter 2014 |
| Intellectual property/ownership of data unclear or compromised | Data application | Mittelstadt and Floridi 2016; Sivarajah et al., 2017 |
| Group-level harms/profiling not considered | Data application | Mittelstadt and Floridi 2016; Zook et al. 2017; Zwitter 2014 |
| Premature or unintentional data destruction | Data deletion | Data not available for processing/research |
